# The holistic management of menopausal symptoms among indigenous women in the Gauteng province

**DOI:** 10.4102/phcfm.v18i1.5108

**Published:** 2026-03-13

**Authors:** Maphefo S. Aphane, Ramadimetja S. Mooa, Molatelo M. Rasweswe

**Affiliations:** 1 Department of Nursing Sciences, Faculty of Health Sciences, University of Pretoria, Pretoria, South Africa; 2 Department of Nursing, Faculty of Health Sciences, University of Limpopo, Polokwane, South Africa

**Keywords:** holistic, management, menopause, menopausal symptoms, indigenous practices, indigenous women, Gauteng province

## Abstract

**Background:**

Menopause is a natural process with exacerbating symptoms for some women. Indigenous practices offer culturally rooted options that need systematic evaluation for safety and efficacy, while hormonal therapy, though effective, carries recognised risks and requires monitoring. An evidence-informed understanding of both approaches is essential for safe, individualised and effective menopausal care.

**Aim:**

To investigate the role of indigenous traditional knowledge practitioners in the holistic management of menopausal symptoms among indigenous women in the Gauteng province, South Africa.

**Setting:**

Selected homes of the participants in the Tshwane district, Gauteng province.

**Methods:**

A qualitative focused ethnography approach utilised purposive and snowball sampling to select 10 indigenous knowledge users and holders and 10 traditional health practitioners. Data were collected through in-depth interviews and non-participant observation and analysed using Brewer’s ethnographic analytical framework with computer-assisted qualitative data analysis software.

**Results:**

*Pitsa* (Pot) as a holistic traditional remedy and cultural existentialism emerged as main themes with three sub-themes. Cultural existentialism is a philosophy that situates menopausal meaning-making and care practices within the framework of cultural identity, heritage and shared traditional knowledge.

**Conclusion:**

The study shows that *Pitsa* is perceived by indigenous women as a cultural practice that supports their holistic menopausal experience. It is valued more for identity, meaning-making and cultural support than for proven clinical effectiveness. Recognising such an indigenous knowledge system in menopausal health discourse is important, alongside further empirical evaluation of its clinical effects.

**Contribution:**

The study highlights the need for understanding and supporting the practices preferred by menopausal women.

## Introduction

Menopause is an inevitable natural ageing process that presents different challenges for each woman.^1^ It is associated with a variety of symptoms that differ in intensity and impact among individuals. Some women experience mild physiological symptoms such as hot flushes, night sweats, mood changes and vaginal dryness.^1^ Others report more severe complications, including joint and muscle discomfort, irritability, cognitive difficulties and pain during sexual intercourse.^2^ Unmanaged menopausal symptoms may escalate to chronic conditions such as cardiovascular disease, osteoporosis, Alzheimer’s disease and other musculoskeletal complications.^3^ Severe menopausal symptoms often drive women to seek healthcare interventions.^4^ However, some research argues that menopause is not an illness and that its symptoms do not require treatment interventions.^4,5^

Conventional management of menopause has predominantly focused on biomedical interventions, particularly hormone replacement therapy (HRT), which has been shown to alleviate many menopausal symptoms through oestrogen supplementation.^6^ However, HRT is not universally effective and carries potential side effects such as vaginal bleeding, thromboembolism, stroke and increased cancer risk.^7^ These limitations, combined with poor accessibility in low- and middle-income countries, including South Africa, often drive indigenous women to seek culturally embedded and more accessible treatment alternatives.^8,9^ Indigenous Knowledge Systems (IKS) offer a holistic approach to health that aligns well with the lived experiences and cultural beliefs of African women. These systems promote physical, emotional, mental and spiritual well-being through culturally relevant practices. Indigenous healthcare is deeply rooted in traditions, rituals and community-based knowledge transmission that supports prevention, treatment and rehabilitation.^10^ For many indigenous African women, Traditional Health Practitioners (THPs) and Indigenous Knowledge Holders (IKHs) serve as trusted sources of information and care.^11^ They are accessible within the community, offer culturally resonant support, and provide individualised care and treatment designed to address the unique problem of each person.^12^

Despite the widespread reliance on IKHs and THPs in menopausal care among African women, there is a paucity of documented evidence regarding the specific methods used, their effectiveness and the holistic nature of the care provided. Existing research and health promotion materials remain predominantly focused on women in high-income countries, often neglecting the socio-cultural realities of women in low-resource settings.^13^ As a result, the management of menopausal symptoms among indigenous women remains inadequately addressed within mainstream healthcare systems. Additionally, African menopausal women continue to endure symptoms in silence, lacking culturally sensitive interventions and anticipatory guidance that could improve their quality of life and prevent more serious health issues in later life.^14^

The management of menopausal symptoms is not only biomedical but also inherently cultural, requiring a nuanced, context-specific and holistic approach. In South Africa, and particularly within the Gauteng province, indigenous African women are among the marginalised groups whose experiences with menopause are under-represented. Many of these women rely on IKS for the holistic management of menopause, incorporating culturally rooted practices that address physical, emotional and spiritual well-being. Rasweswe and Mulaudzi^15^ argue that the management of menopausal symptoms varies across subcultural groups and should be approached through a culturally sensitive lens that respects both individual and collective experiences.

However, there is limited scholarly documentation and understanding of how these traditional practices are applied, their effectiveness, and how they complement or contrast with biomedical approaches. Anticipatory guidance on menopausal symptoms should be reinforced to empower women across diverse contexts to proactively manage their symptoms. The lack of integration and recognition of IKS in formal health frameworks poses a risk of marginalising culturally appropriate care. This gap highlights the need to investigate the role of IKS in managing menopausal symptoms among indigenous women in Gauteng province, to inform culturally sensitive and inclusive health guidelines. In doing so, the study will contribute to the growing body of knowledge advocating for the integration of indigenous practices into national health strategies and the development of culturally responsive care for menopausal women in South Africa.

## Research methods and design

### Study design

This study adopted a qualitative, focused ethnographic design. Focused ethnography was employed to explore specific cultural practices within a well-defined scope, rather than aiming for a broad understanding of an entire culture and its dimensions.^16,17^ The focused ethnographic design enabled the researchers to gather in-depth insights into the holistic and culturally informed management of menopausal symptoms within the Tshwane district, Gauteng province, South Africa.

### Setting

The study was conducted in participants’ homes across various townships in the Tshwane district of Gauteng province. This setting was intentionally selected to provide a familiar, safe and comfortable environment, ensuring confidentiality, privacy and security for participants. Additionally, conducting interviews in their homes proved to be both time-efficient and cost-effective, reducing logistical burdens during data collection, while minimising disruptions to the women’s daily lives.

Gauteng province is renowned for its vibrant cosmopolitan character, blending dynamic urban centres, semi-rural townships and rural communities. This diversity is reflected in its population, which encompasses a rich array of cultural backgrounds, beliefs and traditional practices. Within this multifaceted provincial landscape, the Tshwane district emerges as a significant cultural and demographic hub. With a population of approximately 2.7 million, predominantly of African descent, Tshwane is a melting pot of heritage and modernity.^18,19^

A striking feature of the district is its deep-rooted connection to IKS, exemplified by the estimated 150,000 to 200 000 THPs operating within the region.^20^ These practitioners play a vital role in community health and cultural preservation, offering remedies and spiritual guidance rooted in centuries-old traditions. Many women in Tshwane embrace holistic approaches to well-being, integrating indigenous healing practices with contemporary healthcare. This interplay between tradition and modernity underscores the enduring influence of African spirituality and ancestral wisdom in shaping the district’s social and health landscapes.

### Study population and sampling strategy

The study focused on indigenous knowledge users, holders and THPs within the Tshwane district who were knowledgeable about managing menopause. Eligible participants were required to have at least 1 year of practical experience in addressing menopausal symptoms in women, ensuring they had sufficient expertise and exposure to diverse cases. Those not actively managing menopause or still in training were excluded from participation.

Purposive and snowball sampling techniques were employed to recruit participants for this study. Purposive sampling was initially used to identify indigenous women who were experiencing or had experienced menopause and who were known to be knowledgeable or actively involved in traditional or indigenous approaches to health. This allowed the researchers to deliberately select individuals who could provide rich, relevant and culturally grounded information.

Following this, snowball sampling was used to expand the participant pool by asking the initial participants to refer other women within their social or community networks who met the inclusion criteria. This approach was particularly useful in accessing participants who may not have been easily reachable through formal channels, especially given the private nature of menopause and the culturally embedded knowledge systems involved in its management. Combining these two sampling methods ensured that the study captured a wide range of perspectives while remaining focused on the specific cultural context of the Tshwane district.

Participant recruitment was facilitated through a gatekeeper who communicated the study details to the Head of the Traditional Healers Organisation (THO) in Johannesburg. Following a formal presentation of the study, permission was granted to access a list of local leaders in the Tshwane district who were knowledgeable about the community and its traditional practices. Telephonic negotiations were conducted with these leaders to secure their voluntary participation and to facilitate the identification of additional participants. Snowball sampling was then employed, whereby initial participants referred others who met the inclusion criteria. All participants were contacted telephonically to schedule appointments, and mutually convenient dates, times and venues, primarily participants’ homes, were arranged for the interviews.

### Data collection

The primary data collection methods employed in this study were in-depth face-to-face interviews and non-participant observation. Participants were given the option to participate in either the interview or the observation or both, based on their level of comfort and preference. These techniques were selected to allow for a comprehensive and nuanced understanding of the cultural practices related to the holistic management of menopausal symptoms among women. Prior to data collection, written informed consent was obtained from all participants. With participants’ permission, all interviews were audio-recorded to ensure accurate capturing of information. In addition, the researcher took detailed field notes, documenting verbal and non-verbal cues, contextual factors, emotional expressions, significant events and environmental features observed during the interactions.^21,22^

Open-ended questions and probing techniques were used throughout both interviews and observational sessions. This approach allowed participants to express themselves freely, while also enabling the researcher to explore deeper meanings, clarify responses, and uncover culturally specific practices or beliefs that might not emerge through structured questioning alone. The use of probing ensured that the data collected were rich, context-specific, and aligned with the focused ethnographic methodology. Interview sessions lasted between 30 min and 60 min, depending on the participant’s availability and willingness to share.

Observations were conducted over periods ranging from 1 h to 2 h, allowing the researcher sufficient time to witness relevant practices, interactions or rituals related to menopause management in their natural settings. Although focused ethnography involves a shorter overall immersion, repeated contact with participants through several visits and observation cycles was maintained. This repeated engagement across different days and settings strengthened immersion and allowed refinement of interpretations over time. During observations, both the participants and their surrounding social contexts were closely examined. Attention was paid to how participants engaged with their environments and navigated daily routines related to managing menopausal symptoms. Observations captured verbal and non-verbal behaviours, interactions and cultural practices, offering insight into how IKS were embedded in their lived experiences.^23^ This enriched the data by providing context beyond what was shared during interviews. These complementary data collection techniques ensured a holistic and in-depth exploration of the phenomenon under investigation, aligning with the goals of focused ethnographic research.

Data collection proceeded until data saturation was achieved at participant 15, as indicated by recurring ideas, interpretations, and cultural references across interviews and observation cycles, with no new themes arising. Nevertheless, an additional three IKHs and two THPs were included to verify that sampling neither stopped too early nor continued beyond the point of conceptual redundancy.

### Data analysis

Data obtained from both interviews and observations were analysed and interpreted using Brewer’s^24^ ethnographic analytical framework, complemented by the use of computer-assisted qualitative data analysis (CAQDA) software. This combination enabled a structured yet adaptable approach to managing and interpreting large volumes of qualitative data. The CAQDA software facilitated efficient data organisation, systematic coding, theme identification and the categorisation of emerging patterns.^25^ At the same time, Brewer’s framework provided the analytical lens through which cultural meanings and social contexts were interpreted.^26^

The analysis followed an iterative-inductive process, characterised by a continuous movement between the research questions, the raw data and evolving interpretations. This cyclical engagement enabled the constant refinement of ideas and a deeper interrogation of the data, ensuring that emerging insights were both contextually grounded and theoretically informed. Through this process, the researcher was able to uncover nuanced cultural practices and beliefs embedded in participants’ narratives and behaviours.^27^

Furthermore, this analytical approach encouraged critical reflection and rigorous questioning, enhancing the credibility, depth and cultural sensitivity of the findings. By remaining open to new interpretations and responsive to emerging themes, the researcher maintained analytical flexibility while ensuring methodological rigour throughout the study.^28^

### Trustworthiness

Trustworthiness in this ethnographic research was supported through multiple strategies, including varied data collection methods, purposive sampling, open-ended and neutral phrasing of questions, prolonged engagement with participants, and ongoing reflexivity.^29^ The researcher applied key verification strategies to maintain rigour throughout the study. Investigator responsiveness was achieved by demonstrating openness, active listening and non-judgemental engagement during interviews. Methodological coherence was maintained by aligning the research question, data collection methods and analytical approach. The sampling process ensured the inclusion of participants, IKHs and THPs, who were most knowledgeable and representative of the study context. During data collection and analysis, an iterative process allowed for continuous reflection and alignment between emerging findings and the conceptual framework.^30^ Finally, theoretical thinking was maintained through constant engagement with relevant literature, member checking and the verification of emerging insights, ensuring conceptual depth and analytical consistency.^31^

### Ethical considerations

Ethical approval to conduct the study was obtained from the Faculty of Health Sciences Research Ethics Committee at the University of Pretoria (Protocol number: 528/2020). Additional permissions were secured from the Gauteng Health Research Ethics Committee and the Traditional Healers Organisation (THO). Throughout the research process, strict ethical standards were adhered to protect both the participants and the researcher. Measures were taken to ensure privacy, safety and confidentiality during data collection, particularly during interviews conducted in participants’ homes.^32^ Participants were provided with clear, written and verbal information about the study and their rights, and informed consent was obtained before participation. The study also respected voluntary participation, allowed participants to withdraw at any stage without consequence, and maintained cultural sensitivity throughout engagement with IKHs and THPs. All data were securely stored and anonymised to safeguard participants’ identities.

## Results

A total of 20 women participated in the study. They consisted of 10 IKHs and 10 THPs. From the analysis, two main themes and six sub-themes emerged. The main themes were: (1) *Pitsa* (Pot) as a Holistic Traditional Remedy; and (2) Cultural Existentialism in the Management of Menopause. Table 1 presents the themes and sub-themes.

**TABLE 1:** The themes and sub-themes.

Themes	Sub-themes
1. *Pitsa* as a holistic traditional remedy	1.1. *Pitsa* as the core practice in menopausal management. 1.2. Integration with lifestyle practices. 1.3. Cultural rationale and symbolism.
2. Cultural existentialism in the management of menopause	2.1. Menopause as a cultural transition. 2.2. Identity and belonging through traditional healing. 2.3. Meaning-making and empowerment.

### Theme 1: *Pitsa* as a holistic traditional remedy

*Pitsa* (Pot) refers to a traditional medicinal blend made from a combination of boiled plant roots, leaves, stems, and occasionally insects, prepared for healing purposes. Analysis of the data collected in this study indicated that *Pitsa* is regarded as the most effective indigenous health practice for managing menopausal symptoms. Three key sub-themes supported this theme.

#### Sub-theme 1.1: *Pitsa* as the core practice in menopausal management

*Pitsa* is widely regarded by THPs as a trusted and effective indigenous remedy for managing menopausal symptoms. This multipurpose concoction involves mixing various medicinal plants, leaves and roots collected from fields and mountains to prepare a herbal drink known as *Pitsa*. Participants explained that this traditional remedy is known by several names across different local dialects, including *‘Imbiza’, ‘Mbita’, ‘Dipitsa’*, and *‘Pheko ya madi’*, all of which refer to a mixture of plants and herbs. It is highly esteemed in communities as a key health practice in addressing menopausal challenges.

As one participant explained:

‘You start by giving the “*Dipitsa tsa Sesotho*” for the signs and symptoms that she presents with. Dipitsa is our traditional mixture. You take more than one “*dihlare, medu*” [different types of medicinal plants and roots] or herb, you mix and make *Pitsa*, you cook it, then she drinks it.’ (THP 3, 56 years old, 20 years’ experience)

One preparation and specific ingredients of *Pitsa* vary according to the symptoms presented by the woman. THPs incorporate culturally relevant herbs with targeted purposes such as reducing body heat, inducing sleep, easing joint pain or regulating blood flow.

One participant explained the process, busy preparing the mixture for the client:

‘You see, we give them “*imbiza*.” You will have to make *Imbiza*. When you make it, you add *mathuba difala*, African potatoes, “*hlonya*” [different types of traditional plants and roots]. Sometimes we add a little bit of *“skanama”* [traditional plant], because it makes the stomach to run.’ (TPH 5, 65 years old, 25 years’ experience)

Another participant also uttered that:

‘We make her “*pheko*” [traditional medicine] for blood, the medicine assists with easing the pain and cooling off “heat, anyway, it depends on the symptoms.”’ (THP 2, 70 years old, 20 years’ experience)

One participant emphasised the symptom-specific remedies:

‘We give her *Pitsa* that is going to reduce the heat, when she is not sleeping well and experiences joint pains, I give her that “*setimamollo*” [heat reduction], you add also the other one that stops the pains *“marobadibogale.”*’ (THP 6, 68 years old, 30 years’ experience)

Traditional healing practices for menopause management often incorporate a range of cleansing and therapeutic techniques alongside the use of *Pitsa* to relieve both physical and psychological symptoms. These methods are selected based on the individual’s specific needs.

As shared by one partcipant:

‘We don’t give *Pitsa* alone. She can come sometimes for “*sefotho*” [steaming], “*moaramelo*” [inhalations], “*tsena mbata*” [baths], “*kappa*” [vomit], “*thoba*” [massage], and “*speit*” [enema] depending on the illness.’ (THP 10, 40 years old, 12 years’ experience)

Another participant stated that:

‘We do “*arametsa*” [inhalations], “futha” [steaming], “*kappa*” [vomit], “*speit*” [enema], “*thoba*” [massage], and other techniques.’ (THP 15, 60 years old, 20 years’ experience)

#### Sub-theme 1.2: Integration with lifestyle practices

Culturally rooted dietary practices support the use of *Pitsa* in managing menopause among indigenous African women. The type of food consumed is regarded as a crucial component in managing menopausal symptoms. Traditional Health Practitioners and IKHs commonly advise women to follow specific diets aligned with traditional knowledge systems.

One participant emphasised the role of traditional foods in overall health, saying:

‘Elders were eating “*merogo*” [vegetable plants]. In the past, they consumed a lot of *merogo*, and the meat was not refrigerated. Today we eat meat that has been kept for a long time in the refrigerator, and that causes us problems.’ (THP 7, female, 67 years old, 26 years’ experience)

Similarly, a participant advised against fatty foods and encouraged the consumption of fruits and vegetables and said:

‘Women must avoid fats, and most of the time, they must eat vegetables and fruits, which will help them with these problems.’ (IKH 10, 58 years old, 16 years’ experience)

In addition to diet, physical exercise is also promoted as a key component in the traditional management of menopause. These exercises are not limited to structured routines but include everyday physical activities that support physical, psychological and emotional well-being.

Some participants noted:

‘Indigenous and traditional practices include physical exercises as another method in line with *Pitsa* as their plan of treatment care. This includes walking around, jogging, and doing home duties like laundry and cleaning.’ (IKH 11, 50 years old, 10 years’ experience)

‘Also, to exercise, maybe in the morning, one can walk across the roadside, or maybe clean the house. If you are a woman, you can do the laundry to exercise your body.’ (IKH 1, 62 years old, 15 years’ experience)

A participant noted weight management as another way of assisting *Pitsa* in managing menopause, stating:

‘You need to lose weight and keep your body and muscles flexed, *Pitsa* will work well.’ (IKH 11, 50 years old, 10 years’ experience)

#### Sub-theme 1.3: Cultural rationale and symbolism

The application of *Pitsa* is not only therapeutic but also symbolic, deeply rooted in ancestral teachings and the indigenous cultural understanding of womanhood and healing. It is more than a remedy; it represents a culturally sanctioned pathway to health, especially during menopause, reflecting a holistic view of the female life course within indigenous African communities. The use of *Pitsa* is considered a structured and optimal practice for managing menopausal symptoms. It is often integrated with traditional foods and healing plants to promote recovery and vitality. Participants described how these natural elements work synergistically to support the body’s healing process.

A participant explained:

‘[*T*]hen, when we take this “*medu*” [roots] of our culture and cook like African potatoes, which are the “*magaba*” [traditional potato] and eat, they do something, they build something in our body, then we recover. There are some mountain teas that you drink; there is *“gaolale”* [traditional plant], which is a cultural plant. The woman is supposed to drink the mixture once or twice a day.’ (TPH 1, 57 years old, 27 years’ experience)

Another added that:

‘*Pitsa* assists with mobility until the person gets older and older. If the person continues to drink the *Pitsa* and sometimes also takes traditional plants and roots like “*skanama*”, “*monna wa maledu*” and “*mathuba difala*” cook and drink them, they also clean the body system so that the blood can increase.’ (TPH 11, 60 years old, 25 years’ experience)

Ensuring the effective and rational use of *Pitsa* requires specific qualities such as training, skill and experience. THPs and IKHs undergo traditional training, often complemented by interaction with Western medical practitioners. Moreover, ancestral guidance plays a crucial role in decision-making and treatment planning.

A participant shared:

‘When we train to be traditional practitioners, we go to school where we meet with the Western medicine practitioners, and we teach each other.’ (THP 2, 70 years old, 20 years’ experience)

Another participant emphasised the dual sources of knowledge:

‘I come from the school of the traditional practitioners, but not with my knowledge, but with the direction of the ancestors, the way they instructed me to go and help people out there.’ (THP 3, 56 years old, 20 years’ experience)

She elaborated:

‘We use the education that we received and learned from the school that we went to, together with the education from our ancestors (the spiritual guidance) when they show you how to treat the client.’ (THP 3, 56 years old, 20 years’ experience)

It was noted that not every client is given *Pitsa* indiscriminately. The process involves an initial consultation with spiritual tools, such as bones, to diagnose and tailor treatment to the client’s unique condition. This ensures that the herbs and mixtures used are specifically aligned with the symptoms presented.

A participant clarified:

‘No, it’s not like everyone who comes we give them *Pitsa*. For any client who comes, we consult the bones first because illnesses are not the same. All these herbs that we mix, we first check that they are going to manage the client with the problems that she presents with.’ (THP 19, 64 years old, 18 years’ experience)

### Theme 2: Cultural existentialism in the management of menopause

Cultural existentialism describes the philosophical approaches by which indigenous women root their menopausal experiences, interpretations and coping methods, instead of relying solely on biomedical framing. This theme captures the deep cultural meanings and existential significance that indigenous women in Gauteng attribute to the menopausal transition and its management. This theme is supported by three sub-themes. Rather than viewing menopause solely as a biological or medical event, participants described it as a cultural milestone marking a shift in identity, purpose and social role. Menopause was understood through a lens of spirituality, ancestral connection and womanhood, with traditional healing practices such as the use of *Pitsa* serving as both therapeutic and symbolic acts. Engaging in these practices enabled women to find meaning, maintain their cultural identity, and assert autonomy over their bodies and health in ways that aligned with their world views. This culturally rooted interpretation of menopause provided not just symptom relief, but emotional strength, spiritual affirmation and a sense of belonging.

#### Sub-theme 2.1: Menopause as a cultural transition

Among indigenous African communities, menopause is not perceived solely as a biological event but as a profound spiritual and existential shift. It is marked by cultural rituals and the use of *Pitsa*, which holds both healing and symbolic significance. This phase of life is often embraced with respect, representing a transition to a new identity and role within society.

As one participant noted:

‘Menopause is not just about the body changing; it is a spiritual journey that every woman must go through with respect and care.’ (IKH 4, 57 years old, 14 years’ experience)

Participants explained that menopause is considered a culturally significant life transition. It is often accompanied by traditional rituals, spiritual preparation and guidance from elders, all of which serve to honour the woman’s passage into a new stage of life. These ceremonies are essential in preparing the woman emotionally and spiritually.

One participant shared:

‘The rituals we perform during menopause help us honour this new stage of life and prepare our spirits for the next chapter.’ (TPH 8, 67 years old, 20 years’ experience)

The use of *Pitsa* during menopause extends beyond its medicinal role. It is part of a sacred tradition that links women to their ancestral lineage, reinforcing cultural identity and spiritual strength. Its application is deeply symbolic, reflecting a woman’s connection to her heritage and inner power.

According to one participant:

‘Using *Pitsa* during menopause is part of a sacred tradition that connects me to my ancestors and strengthens my soul.’ (IKH 6, 68 years old, 23 years’ experience)

Participants emphasised that menopause marks a shift from reproductive capacity to a new role as a spiritual elder, adviser, and custodian of wisdom within the family and community. The ceremonies and teachings associated with this stage reaffirm the woman’s status and responsibility in society.

A participant explained:

‘Menopause marks a passage where we become keepers of wisdom, and the ceremonies remind us of our important role in the family.’ (TPH 9, 72 years old, 30 years’ experience)

Rather than viewing menopause through a lens of loss or decline, participants described it as a stage of empowerment and maturity. The transition is viewed as an opportunity to adopt a renewed identity as a culturally respected and spiritually grounded woman.

One participant stated:

‘This transition is more than physical, it is about embracing my identity as a mature woman within our culture.’ (TPH 5, 65 years old, 25 years’ experience)

#### Sub-theme 2.2: Identity and belonging through traditional healing

Traditional healing practices, such as the use of *Pitsa*, are not only health-related interventions but also deeply symbolic expressions of identity among indigenous women. These practices connect women to their ancestral past and cultural heritage. As they engage in preparing and using *Pitsa*, they reaffirm their place within a long-standing cultural lineage.

This link is significant during life transitions such as menopause: ‘Using *Pitsa* connects me to my ancestors; it reminds me of who I am and where I come from’ (IKH 5, female, 56 years old, 8 years’ experience); capturing how the act of traditional healing reinforces a woman’s sense of self and origin.

Healing practices also foster a profound sense of belonging by connecting women to the generations before and after them. The intergenerational aspect of traditional healing empowers women to see themselves as part of a continuous cultural thread. When they engage in rituals and prepare traditional mixtures, they often recall how their mothers and grandmothers did the same. One participant shared: ‘When I prepare and use these traditional mixtures, I feel part of a long line of women who have cared for our community’ (THP 2, 70 years old, 20 years’ experience), emphasising the communal and familial value attached to these practices.

Beyond individual experience, traditional healing serves as a means of cultural continuity and preservation. The use of *Pitsa* allows women to actively preserve IKS that risk being forgotten. By practising and teaching these traditions, they help sustain cultural identity within the broader community. A participant noted: ‘*Pitsa* is not just medicine; it is a way to keep our culture alive and pass it on to the younger generation’ (IKH 6, 68 years old, 23 years’ experience), highlighting the importance of transmission of knowledge and cultural preservation.

Traditional healing also supports emotional and psychological well-being. It grounds women spiritually, particularly during emotionally vulnerable periods such as menopause. These practices validate their experience and offer culturally appropriate forms of support.

A participant explained: ‘Through these healing practices, I feel strong and grounded in my identity as an indigenous woman’ (THP 3, 56 years old, 20 years’ experience). This groundedness provides emotional resilience and strengthens cultural pride.

Ultimately, the communal and ritualistic aspects of traditional healing promote inclusion and social recognition. During rituals surrounding *Pitsa*, women feel acknowledged and respected within their cultural setting. These moments mark their transition into a new role within the family and community, often one of leadership and wisdom. A participant said: ‘The rituals around *Pitsa* make me feel included, respected, and proud of my heritage, especially during menopause’ (TPH 9, 72 years old, 30 years’ experience), illustrating how these experiences enhance both individual confidence and communal status.

#### Sub-theme 2.3: Meaning-making and empowerment

The use of traditional mixtures, such as *Pitsa*, is deeply embedded in the cultural identity of indigenous women. More than a remedy, *Pitsa* serves as a symbolic bridge to their ancestry, anchoring them to generations of indigenous wisdom and healing. Engaging in these practices fosters a profound spiritual connection that reinforces both personal and communal identity.

As one participant reflected: ‘Using *Pitsa* connects me to my ancestors; it reminds me of who I am and where I come from’. (IKH 5, female, 56 years old, 8 years of experience)

For most participants, the preparation and use of *Pitsa* affirm their place within a historical and cultural lineage of women healers and caregivers. This recognition strengthens their sense of purpose and responsibility in preserving cultural knowledge and ensuring its transmission to future generations.

One of the participants expressed this connection clearly: ‘When I prepare and use these traditional mixtures, I feel part of a long line of women who have cared for our community’. (THP 7, female, 67 years old, 26 years’ experience)

*Pitsa* is not viewed merely as medicinal, but as a means to keep IKS alive. Through its continued use, women contribute to the survival and relevance of their cultural practices in the face of modernisation and potential loss of tradition.

A participant noted: ‘*Pitsa* is not just medicine; it is a way to keep our culture alive and pass it on to the younger generation’. (IKH 6, 68 years old, 23 years’ experience)

Participating in these traditional healing practices offers emotional and spiritual strength. It provides women with a renewed sense of self and resilience, particularly during the transformative phase of menopause. Being grounded in their identity offers a source of empowerment and pride.

One participant affirmed: ‘Through these healing practices, I feel strong and grounded in my identity as an indigenous woman’ (THP 4, female, 60 years old, 14 years’ experience).

The rituals surrounding *Pitsa* foster a sense of inclusion and recognition. Women felt acknowledged and respected within their cultural framework, especially during the often-challenging period of menopause. These traditions reinforce their social significance and collective pride.

A participant shared: ‘The rituals around *Pitsa* made me feel included, respected, and proud of my heritage, especially during that time of menopause’. (IKH 8, female, 78 years old, 30 years’ experience).

## Discussion

The study explored the holistic management of menopausal symptoms among indigenous women in the Gauteng province. The findings highlight the centrality of *Pitsa*, a traditional herbal preparation made from boiling various plant roots, stems, leaves, and occasionally insects as a culturally embedded remedy for managing menopausal symptoms in indigenous African communities. The findings affirm the prominence of *Pitsa* as an indigenous therapeutic intervention grounded in holistic health principles that address not only the physiological changes of menopause but also its psychosocial and spiritual dimensions. This aligns with the literature on African indigenous medicine, which emphasises that healing involves the restoration of balance within the individual and between the individual and their ancestors, environment and community.^33,34^ Moreover, traditional herbalism is deeply intertwined with indigenous identity, spiritual beliefs and communal knowledge transmission, suggesting that preparations like *Pitsa* function as both curative agents and cultural artefacts.^35,36^ The findings affirm the value of *Pitsa* beyond biomedicine and advocate for its respectful inclusion in health dialogues and frameworks.

Traditional health practitioners regard *Pitsa* as a foundational treatment modality for menopause-related symptoms. The remedy is crafted using a variety of local medicinal plants, selected based on the presenting symptoms, with each ingredient believed to possess specific therapeutic properties. For instance, herbs are included to alleviate internal heat, ease joint pain, regulate blood flow, or promote sleep. This individualised approach reflects the adaptive and symptom-specific nature of traditional African healing systems, where practitioners draw upon extensive biocultural knowledge to guide their interventions.^37,38^ Moreover, *Pitsa* is rarely administered in isolation. It is typically complemented by other therapeutic practices, including steaming (*sefotho*), inhalations (*moaramelo*), medicinal baths (*tsena mbata*), enemas (*speite*), vomiting (*kapa*) and massage (*thoba*). These practices form a holistic treatment protocol tailored to the physical, emotional and spiritual needs of the individual. Such integrative techniques are consistent with indigenous frameworks of health, which prioritise balance and harmony within the individual and their broader environment.^39^ The use of *Pitsa* as a core remedy reflects the broader indigenous conceptualisation of menopause not solely as a biomedical event but as a transition requiring a multi-layered response. This aligns with the literature that acknowledges the limitations of pharmacological approaches and calls for integrative care models that accommodate diverse cultural beliefs and practices.^40^

The therapeutic efficacy of *Pitsa* in managing menopause among indigenous African women is not seen as isolated but is embedded within a broader ecosystem of culturally grounded lifestyle practices, particularly diet and physical activity. Traditional Health Practitioners and IKHs emphasise that *Pitsa* is most effective when used in conjunction with specific dietary modifications, including the consumption of indigenous vegetables such as *merogo* (wild leafy greens), unprocessed meats, fruits, and low-fat, high-fibre foods. These dietary prescriptions are not merely pragmatic but reflect a cosmological and ecological view of health, where food is both nourishment and medicine. Rasweswe and Mulaudzi^15^ highlighted how such indigenous dietary practices are integral to menopause management in African contexts, promoting metabolic balance, blood purification and spiritual cleanliness. This indigenous epistemology aligns with global nutritional science, which recognises the role of whole, plant-based foods in reducing menopausal symptoms and promoting overall well-being.^41,42^

In addition to diet, physical activity forms a crucial pillar of traditional menopausal care. However, unlike Western paradigms that emphasise structured exercise regimes, indigenous approaches are embedded in everyday life, walking to fetch water, sweeping yards, doing laundry and engaging in agricultural tasks. These practices not only promote physical health but also maintain social roles and foster a sense of purpose and continuity. Rasweswe and Mulaudzi^15^ illustrate that these daily movement routines serve both functional and therapeutic purposes, and are understood by THPs as activating circulation, improving joint function, and aiding detoxification. Such views are consistent with recommendations by the World Health Organization, which endorses regular physical activity as a non-pharmacological intervention for managing vasomotor symptoms and improving quality of life during menopause.^43^

Together, these practices, such as herbal therapy, culturally informed diets and embedded physical activity, form an integrative, systems-based approach to health that is deeply aligned with indigenous world views. This model affirms the body-environment-spirit nexus, which is foundational to African healing systems, echoing the same principles.^44,45^ It is noted that indigenous medicine globally operates through interconnected and holistic principles rather than reductionist biomedical frameworks. Hence, *Pitsa* must be understood not as an isolated remedy but as a key component in a web of healing traditions that collectively enable cultural coherence, spiritual affirmation and embodied resilience during the menopausal transition.

The use of *Pitsa* transcends its physical constituents to embody rich cultural and symbolic meanings. It is deeply rooted in ancestral knowledge systems and represents a culturally sanctioned path towards healing and maturity. Within many African communities, menopause is not only a physiological transition but also a spiritual milestone, marking the entrance into elderhood and often associated with increased spiritual authority and communal respect.^46,47^ The preparation and administration of *Pitsa* are considered sacred acts guided by ancestral direction. Traditional health practitioners often receive their training through spiritual means, such as dreams, visions and ritual initiation, and combine this knowledge with formal engagements, including collaborative interactions with biomedical professionals. This dual epistemology challenges binary distinctions between traditional and biomedical systems, reflecting a growing recognition of hybrid knowledge formations in contemporary African health practices.^48,49^

Moreover, THPs do not administer *Pitsa* indiscriminately. The treatment process involves an initial diagnostic consultation, often utilising spiritual tools such as *ditaola* (bones), to determine the appropriateness and composition of the remedy. This reinforces the principle of individualised care and reflects indigenous methodologies of intuitive diagnostics, which prioritise the uniqueness of each patient’s physiological and spiritual profile.

The symbolic potency of *Pitsa* is further amplified by its deep association with natural elements, such as roots, plants and underground tubers, which are believed to embody ancestral power and mediate processes of physical and spiritual renewal. These botanical elements are regarded not merely as pharmacologically active substances but as sacred conduits that purify the body, restore internal balance, and reconnect women with the cyclical rhythms of nature. During transitional life phases, such as menopause, these natural substances are believed to realign the body with the cosmos and fortify the woman’s spiritual agency. This interpretation is consistent with the World Health Organization (2014–2023), which recognises that traditional medicine in many African contexts transcends symptom management to engage with spiritual, social and cosmological dimensions of health.^50,51^ Furthermore,^39,52^ it is argued that indigenous medicinal practices are often embedded in ritual and symbolism, with healing processes reflecting community values, ancestral ties and metaphysical beliefs rather than merely biomedical rationales. Together, these perspectives affirm that *Pitsa* operates within a framework where medicine is ritual, and healing is both a physical and spiritual act deeply interwoven with identity, belonging and cultural continuity.

Furthermore, the findings showed that the management of menopause among indigenous women in Gauteng transcends biomedical framings, instead reflecting a deeply existential and culturally embedded experience. Participants constructed menopause not simply as a physiological cessation of menstruation but as a culturally recognised milestone of transformation. The transition into menopause is marked by rituals, traditional healing and spiritual realignment, situating it as a process of identity reconstruction and social repositioning within one’s community. Thus, indigenous African conceptualisations of menopause are imbued with spiritual and social meaning. Rather than viewing menopause as a medicalised pathology to be treated or mitigated, participants articulated it as a spiritual journey requiring preparation, respect and guidance. Traditional healing rituals, including the preparation and ingestion of *Pitsa*, function as both therapeutic interventions and rites of passage. This symbolic framing aligns with anthropological literature that recognises life stage transitions such as birth, puberty and menopause as culturally inscribed events often marked by ceremonial practices.^53,54^

The women’s narratives indicate that the menopausal transition initiates a redefinition of identity. This phase heralds a shift from reproductive roles towards becoming spiritual elders, advisers and custodians of familial wisdom. Such transitions confer a heightened social status rather than precipitating decline or invisibility, as often reported in Western biomedical or feminist discourse.^55^ The cultural positioning of post-menopausal women as repositories of moral and spiritual authority highlights a counter-narrative to the dominant pathologising of menopause, foregrounding it instead as a time of empowerment and respect. *Pitsa*, in this context, is not merely a phytotherapeutic agent but a medium for spiritual purification and ancestral alignment. Its administration is accompanied by rituals that serve to recalibrate the woman’s place in her family, lineage and cosmos. These practices reflect broader African cosmologies, which perceive health as a state of harmony between the physical, spiritual and ancestral realms.^56,57^ Hence, menopause emerges as a liminal stage, requiring sacred acts of reintegration and transformation.

Traditional healing practices are not only curative, but they also serve as cultural affirmations of selfhood and a sense of belonging. The repeated reference to *Pitsa* as being prepared using indigenous plants and ancestral guidance reveals its dual role as both a medicinal substance and a mnemonic device. For many participants, the act of preparing and consuming *Pitsa* evoked memories of mothers and grandmothers, positioning it as a site of intergenerational connection and cultural continuity. This phenomenon illustrates the principle of ‘embodied heritage’, whereby cultural identity is enacted and sustained through everyday practices.^58,59^ The use of traditional medicine during menopause thus reaffirms the woman’s place in a lineage of caregivers and healers. Such healing rituals are performative acts of identity that ground women in their historical and cultural milieu. As they perform rituals learned from their elders, women become active agents in the transmission of indigenous knowledge, a critical process given the threats posed by modernisation, urbanisation and biomedical dominance (WHO 2014–2023).^39,50^ The transmission of healing knowledge becomes a political and epistemological act, resisting erasure and asserting legitimacy in pluralistic health landscapes.

The communal aspects of traditional healing, where rituals are shared, witnessed, and sanctioned by others, foster a sense of belonging and social validation. Ritual participation, especially during menopause, marks a public recognition of the woman’s evolving role, reinforcing both individual self-worth and communal cohesion. The women’s narratives illustrate how ritual engagement mediates psychosocial well-being, offering culturally sanctioned avenues for emotional resilience and existential affirmation.^60,61^

Engaging in traditional healing practices such as the use of *Pitsa* enabled women to find meaning in their menopausal journey and articulate an empowered sense of self. The preparation and use of *Pitsa* provided more than symptom relief; it offered a spiritual grounding and a framework through which women could interpret their changing bodies within culturally coherent narratives. This sense-making process is crucial to resilience, particularly during life transitions marked by uncertainty or loss.^62,63^

The cultural rituals surrounding menopause allowed women to reclaim authority over their bodies and experiences, countering narratives that associate ageing with decline. Participants expressed a renewed sense of pride in their heritage, confidence in their knowledge, and responsibility to pass this wisdom to future generations. This reflects the feminist principle of ‘reclaiming the body’^64,65^ yet situated within an indigenous paradigm that views the body not as a site of struggle, but as a vessel for spiritual power and cultural expression. Moreover, the role of *Pitsa* as a symbol of resistance against cultural erosion was especially salient. In invoking and practising ancestral methods, women were not only healing themselves but also asserting the continued relevance and authority of IKS. This aligns with the literature that emphasises the decolonial imperative to recognise and restore epistemologies marginalised by colonial and Western biomedical hegemonies.^66,67^ Traditional healing thus becomes a form of epistemic resistance and cultural affirmation, positioning indigenous women as active custodians of living knowledge systems.

### Recommendations

Based on the insights gained from this study, the authors recommend formal recognition of indigenous health frameworks and greater collaboration with THPs, particularly in community-based women’s health programmes. In addition, health education and training curricula should include modules that sensitise health professionals to the socio-cultural and spiritual dimensions of menopause as experienced by diverse populations. This will cultivate cultural humility and enable practitioners to better align care with the values and needs of indigenous women. Furthermore, policymakers should support the integration of indigenous perspectives into national health policies, including funding for community-led menopause education and cultural wellness programmes. Finally, further interdisciplinary research should explore how indigenous epistemologies can inform broader frameworks of women’s health, ageing and well-being. Such research should be conducted in partnership with indigenous communities, ensuring that knowledge generation is reciprocal, respectful and mutually beneficial to both academic and community interests.

### Strengths and limitations

The findings of this study have significant implications for health research, education and practice, particularly in the context of culturally diverse and historically marginalised populations. They highlight the centrality of IKS in shaping health experiences and the need to move beyond biomedical paradigms that often universalise menopausal transitions. The deeply symbolic, spiritual and culturally embedded ways in which indigenous women understand and manage menopause highlight that health cannot be divorced from cultural identity, social positioning and spiritual meaning. Health practitioners, particularly those operating in multicultural settings, must therefore engage with culturally specific world views and healing practices if they are to provide care that is not only clinically effective but also socially and spiritually responsive. Moreover, the findings emphasise the importance of recognising menopause as a socially valorised transition rather than a biomedical deficit, offering a counter-narrative to dominant discourses that frame ageing and reproductive cessation in terms of loss or dysfunction. This re-framing has implications for how health education materials, clinical consultations, and policy frameworks conceptualise and communicate about women’s health in midlife.

The limitations of the study are that findings cannot be generalised to a broader population, as it was conducted in the specific district of Gauteng province. Only one district was used to gather data to contextualise the study. Although the conclusions of the study cannot be generalised, they can be used in other contexts to enhance the implementation of holistic management of menopause.

## Conclusion

This study shed light on the multifaceted and culturally grounded understandings of menopause among indigenous women in Gauteng, South Africa. Through an interpretive lens grounded in ethnography, menopause was revealed as a dynamic, socially embedded and spiritually meaningful life transition. The findings highlighted the primacy of indigenous knowledge in shaping the interpretation and meaning of menopausal experiences. The findings also emphasised the cultural and existential practices through which menopause is navigated and managed. The traditional healing practices, especially the ritualistic use of *Pitsa*, play a critical role in enabling women to make meaning of their menopausal journey, reaffirm identity and enact belonging.

Collectively, the findings affirm that IKS offer coherent and holistic frameworks that support psychological well-being, social recognition and cultural continuity during menopause. They also challenge the prevailing clinical narratives that dominate global health discourses, urging greater acknowledgement of culturally situated experiences and epistemologies. As a contribution to decolonial scholarship and culturally responsive health research, this study advocates for the inclusion of indigenous knowledge in the design of menopause education, support programmes and health policy. Recognition of traditional practices and world views rather than their marginalisation can enhance the dignity, agency and health of indigenous women as they transition through menopause. Future research and health interventions should thus be grounded in epistemic humility, engaging respectfully with IKHs and community structures to co-create models of care that resonate with cultural meaning and lived realities.
